# Acute Kidney Injury Associated with Synthetic Cannabinoid Use — Multiple States, 2012

**Published:** 2013-02-15

**Authors:** Tracy D. Murphy, Kelly N. Weidenbach, Clay Van Houten, Roy R. Gerona, Jeffery H. Moran, Ronald I. Kirschner, Jeanna M. Marraffa, Christine M. Stork, Guthrie S. Birkhead, Andie Newman, RobertG. Hendrickson, B. Zane Horowitz, Karen Vian, Richard F. Leman, Stephen L. Thornton, Clayton Wood, David A. Myers, Erik Orr, John J. Devlin, Michael D. Schwartz, Genevieve L. Buser

**Affiliations:** Wyoming Dept of Health; Dept of Laboratory Medicine, Univ of California–San Francisco; Arkansas Public Health Laboratory, Arkansas Dept of Health; Nebraska Regional Poison Center; Upstate Medical Univ, Upstate New York Poison Center; New York State Dept of Health; Oregon Poison Center; Douglas County Public Health; Oregon Public Health Div; Univ of Kansas Hospital Poison Control Center; Dept of Emergency Medicine, Univ of Kansas Hospital; Oklahoma Univ Health Sciences Center; Geospatial Research, Analysis, and Services Program; Office of Environmental Health Emergencies, National Center for Environmental Health; EIS Officer, CDC

In March 2012, the Wyoming Department of Health was notified by Natrona County public health officials regarding three patients hospitalized for unexplained acute kidney injury (AKI), all of whom reported recent use of synthetic cannabinoids (SCs), sometimes referred to as “synthetic marijuana.” SCs are designer drugs of abuse typically dissolved in a solvent, applied to dried plant material, and smoked as an alternative to marijuana. AKI has not been reported previously in users of SCs and might be associated with 1) a previously unrecognized toxicity, 2) a contaminant or a known nephrotoxin present in a single batch of drug, or 3) a new SC compound entering the market. After the Wyoming Department of Health launched an investigation and issued an alert, a total of 16 cases of AKI after SC use were reported in six states. Review of medical records, follow-up interviews with several patients, and laboratory analysis of product samples and clinical specimens were performed. The results of the investigation determined that no single SC brand or compound explained all 16 cases. Toxicologic analysis of product samples and clinical specimens (available from seven cases) identified a fluorinated SC previously unreported in synthetic marijuana products: (1-(5-fluoropentyl)-1H-indol-3-yl)(2,2,3,3-tetramethylcyclopropyl) methanone, also known as XLR-11, in four of five product samples and four of six patients’ clinical specimens. Public health practitioners, poison center staff members, and clinicians should be aware of the potential for renal or other unusual toxicities in users of SC products and should ask about SC use in cases of unexplained AKI.

## Epidemiologic Findings

The first three patients (Table 1, cases 1–3) reported smoking SCs in the days or hours before symptom onset. Public health staff members interviewed the three and reviewed their medical records. The patients were young, previously healthy males who reported smoking either a blueberry-flavored SC product (one patient) or an unspecified SC product (two patients). They experienced severe nausea, vomiting, and flank or abdominal pain and went to emergency departments during February 26–29. Local law enforcement officials were notified and released a media advisory warning of illness associated with SC use.

The Wyoming Department of Health launched an investigation to identify other cases and determine the cause of illness. A case initially was defined as nausea, vomiting, abdominal or back pain, and AKI (i.e., serum creatinine concentration above the facility’s reference range) in a patient reporting SC use and illness onset during February 1–March 1. Hospital staff members from two regional medical facilities conducted retrospective reviews of emergency department and hospital admission records. The Wyoming Department of Health issued a health alert on March 1 to all licensed health-care providers, hospitals, emergency departments, and urgent-care centers in Wyoming, describing the possible association between AKI and SC use and requesting that potential cases be reported. On March 21, the Wyoming state epidemiologist contacted CDC regarding the first three cases. On March 24, a fourth Wyoming patient became ill after smoking either a blueberry-flavored or bubblegum-flavored SC product and was found to meet the case definition (Table 1, case 4).

A collaboration among several state public health officials, poison center toxicologists, forensic laboratory scientists, individual clinicians, and the Arkansas K2 Research Consortium, identified an additional 12 cases of SC-associated AKI in Oregon (six cases), New York (two), Oklahoma (two), Rhode Island (one), and Kansas (one) in hospitalized patients who had serum creatinine concentration above the facility’s reference range after smoking an SC product during March 16–December 3. CDC medical toxicologists reviewed clinical and laboratory data from all 16 cases (Table 1).

All 16 patients initially visited emergency departments and subsequently were hospitalized. The 16 patients included 15 males aged 15–33 years (median: 18.5 years) and one female aged 15 years; all but one had nausea and vomiting. Twelve patients reported abdominal, flank, and/or back pain. None reported preexisting renal dysfunction or use of medication that might have caused renal problems. The highest serum creatinine concentrations (creatinine peak) among the 16 patients ranged from 3.3 to 21.0 mg/dL (median: 6.7 mg/dL; normal 0.6–1.3 mg/dL) and occurred 1–6 days after symptom onset (median: 3 days). Urinalysis for 15 patients showed variable results: proteinuria (eight patients), casts (five), white blood cells (nine), and red blood cells (eight). Twelve patients underwent renal ultrasonograpy, nine of whom had a nonspecific increase in renal cortical echogenicity; none had hydronephrosis.

Six of eight patients with a renal biopsy demonstrated acute tubular injury, and three of eight patients demonstrated features of acute interstitial nephritis. Kidney function recovery was apparent within 3 days of creatinine peak in most patients. However, five of the 16 patients required hemodialysis, and four patients received corticosteroids; none died. Other infectious, autoimmune, pharmacologic, or other toxic causes of AKI were not found.

## Toxicologic Analysis

Of the 16 cases, toxicologic analysis of implicated SC products and clinical specimens was possible in seven (Table 2). No single SC product explained all of the cases. Two SC products recovered by law enforcement officials in Wyoming and epidemiologically linked to cases 1–3 were tested by the Arkansas K2 Research Consortium laboratory (Arkansas K2) and the University of California–San Francisco Clinical and Environmental Toxicology Laboratory (UCSF). Gas chromatography/mass spectrometry (Arkansas K2) and liquid chromatography/time-of-flight mass spectrometry (UCSF) analysis revealed that both products contained 3-(1-naphthoyl) indole, a precursor to several aminoalkylindole synthetic cannabinoids. One of the two product samples also contained the potent synthetic cannabinoid AM2201, which has been linked to human disease and death, but not to AKI.

Standardized liquid chromatography–time of flight mass spectrometry methods validated for trace level analysis of synthetic cannabinoid parent compounds and metabolites were used for all clinical assays (UCSF). A sample of the product smoked by the patient in case 4 contained 3-(1-naphthoyl) indole and XLR-11, a previously undescribed fluorinated-derivative of the SC compound UR-144 currently in circulation. A urine specimen collected from the same patient was positive for the XLR-11 N-pentanoic acid metabolite. A blood specimen from the patient in case 6, who smoked “Phantom Wicked Dreams,” contained the N-pentanoic acid metabolite of XLR-11. In case 11, analysis of the SC product “Mr. Happy” and a serum specimen revealed the SCs XLR-11 and UR-144; a urine specimen contained the N-pentanoic acid metabolite of XLR-11. In case 12, samples of “Clown Loyal” were found to contain XLR-11. In cases 13 and 14, analysis of “Lava” and associated clinical specimens identified XLR-11 and the N-pentanoic acid metabolite of XLR-11. In case 15, analysis of “Flame 2.0” was negative for XLR-11. For nine of the 16 cases, neither product samples nor clinical specimens were available for analysis.

What is already known on this topic?

Synthetic cannabinoids (SCs) are psychoactive chemicals dissolved in solvent, applied to plant material, and smoked as a drug of abuse. They are sold in “head shops” and tobacco and convenience stores under labels such as “synthetic marijuana,” “herbal incense,” “potpourri,” and “spice.” Most reports of adverse events related to SCs have been neurologic, cardiovascular, or sympathomimetic.

What is added by this report?

Sixteen cases of acute kidney injury following exposure to SCs were identified in six states with illness onset during March 16– December 7, 2012. Patients ranged in age from 15 to 33 years; 15 were male, and none reported a history of kidney disease. Gas and liquid chromatography and mass spectrometry identified a new SC, XLR-11, associated with some of these cases.

What are the implications for public health practice?

Novel drugs of abuse are emerging continuously. SCs often are packaged in colorful wrappers bearing labels such as “not for human consumption” or “incense,” although health professionals and legal authorities know these products are smoked like marijuana. Law enforcement officials, public health officials, clinicians, scientists, and the members of the public should be aware of the potential for adverse health effects posed by SCs.

### Editorial Note

Synthetic cannabinoid compounds originally were developed to facilitate study of cannabinoid receptor pharmacology, but in recent years have emerged as drugs of abuse. In 2005, SC products marketed as “Spice” first emerged in European countries, before appearing in the United States in 2009, where they were marketed initially as “K2.” Today, SC products are distributed worldwide under countless trade names and packaged in colorful wrappers designed to appeal to teens, young adults, and first-time drug users (1). Products often are packaged with disingenuous labels such as “not for human consumption” or “incense,” but health professionals and legal authorities are keenly aware that these products are smoked like marijuana. Despite federal and state regulations to prohibit SC sale and distribution, illicit use continues, and reports of illness are increasing (1–4).

The expectation of a more intense high than that induced by marijuana, easy access, affordability, and avoidance of detection by many commonly used urine drug tests all contribute to the growing abuse of SCs, especially among male adolescents (1,5). The increasing use of SCs by young persons, coupled with mounting evidence of adverse health effects, has led to state and federal legislation (3,6). However, full recognition of the potential dangers of SCs is not widespread among users or sellers, and SC products remain available on the Internet and at many convenience stores. Further, differences in state drug enforcement statutes have led to different laws and approaches to drug enforcement (7).

Although related to delta-9-tetrahydrocannabinol, the active ingredient in marijuana, SCs are two to three times more likely to be associated with sympathomimetic effects (i.e., tachycardia and hypertension), and approximately five times more likely to be associated with hallucinations (8). In addition, an increase in the occurrence of seizures has been reported with SC use (9). This report describes unanticipated AKI with SC abuse. Given the rapidity with which new SC compounds enter the marketplace and their increasing use in the past 3 years, outbreaks of unexpected toxicity associated with their use are likely to increase.

Management of suspected SC toxicity is symptomatic and supportive; no antidote exists. All of the patients in this report recovered creatinine clearance during their hospital stay, although the length of time was variable; one patient was discharged before his creatinine normalized. However, a risk for long-term kidney sequelae might exist. Recent studies suggest an increased risk for chronic and end-stage renal disease following AKI of various etiologies, despite initial recovery (10). Physicians caring for otherwise healthy adolescents and young adults with unexplained AKI should inquire about SC use, and cases of suspected SC poisoning should be reported to both the regional poison center and the appropriate state health department. Regional poison centers can be reached by telephone at 1-800-222-1222, from anywhere in the United States.

In this report, the product used by five of the 16 patients, including two patients (cases 13 and 14) who used the same product, contained a novel fluorinated SC (XLR-11). In addition, XLR-11 and/or XLR-11 metabolites were found in five of the seven cases for whom clinical specimens were available. XLR-11 emerged on the SC market in the first half of 2012; therefore, experience with this fluorinated compound has been limited. The consistent finding of XLR-11 in product samples and clinical specimens has alternative explanations. XLR-11, a metabolite, or a contaminant associated with it might be responsible for AKI in these patients, or its presence might simply reflect the widespread use of this particular compound in SC products during the study period rather than a causal association with AKI. Health-care providers should be aware of renal and other unexpected toxicities from use of SC products, especially with newer SC compounds.

## Figures and Tables

**TABLE 1:** Demographic and clinical characteristics and implicated product in 16 cases associated with synthetic cannabinoid use — multiple states, 2012

Case no.	State	Patient age (yrs)	Chief symptom at presentation	Peak creatinine (mg/dL)	Urine microscopy results*	Renal ultrasound results	Implicated product
1	Wyoming	19	Nausea and vomiting, abdominal pain	5.2	WBCs, RBCs, RBC/granular casts	Within normal limits	Synthetic cannabinoid, not otherwise specified
2	Wyoming	15	Nausea and vomiting, abdominal pain	6.8	WBCs, RBCs, RBC/ granular casts, eosinophils	Increased cortical echogenicity bilaterally	Synthetic cannabinoid, not otherwise specified
3	Wyoming	21	Nausea and vomiting, flank pain	6.3	WBCs, RBCs, epithelial casts, granular casts	Not available	Blueberry-flavored
4	Wyoming	18	Nausea and vomiting, flank pain	4.1	Hyaline casts, WBCs	No increased cortical echogenicity or hydronephrosis	Blueberry-flavored or bubblegum-flavored
5	Rhode Island	25	Nausea and vomiting, anuria	21.0	RBCs, proteinuria, eosinophils	Not performed	Synthetic cannabinoid, not otherwise specified
6	New York	30	Nausea and vomiting	9.0	WBCs, RBCs, RBC/ hyaline casts,	Not performed	Phantom Wicked Dreams
7	Oregon	18	Nausea and vomiting, abdominal pain pain	6.6	WBCs, protein 30	Increased cortical echogenicity, no hydronephrosis	“Synthetic marijuana”
8	New York	33	Nausea and vomiting	3.3	Not available	Not performed	Spice Gold
9	Oregon	27	Flank pain	4.7	Small blood, protein 30	Normal echogenicity, no hydronephrosis	Mad Monkey or Clown Loyal
10	Washington/Oregon	15	Nausea and vomiting, abdominal pain / back pain	9.1	Protein trace	Increased cortical echogenicity, no hydronephrosis	Synthetic cannabinoid, not otherwise specified
11	Kansas	26	Nausea and vomiting, abdominal pain / back pain	7.7	Within normal limits	Increased cortical echogenicity	Mr. Happy
12	Oregon	17	Nausea and vomiting, flank pain	10.6	WBCs, RBCs, protein 2+, eosinophils 1+	Increased cortical echogenicity, no hydronephrosis	Clown Loyal
13	Oregon	18	Nausea and vomiting, abdominal pain pain	9.6	Protein 2+, blood 3+, no RBCs	Increased cortical echogenicity, no hydronephrosis	Lava
14	Oregon	18	Nausea and vomiting, abdominal pain pain	5.5	Protein 1+	Increased cortical echogenicity, no hydronephrosis	Lava
15	Oklahoma	15	Nausea and vomiting, abdominal pain pain	11.5	WBCs, RBCs	Increased cortical echogenicity, bilateral symmetrical enlargement	Flame 2.0
16	Oklahoma	15†	Nausea and vomiting	6.2	WBC, protein 1+	Increased cortical echogenicity	Flame 2.0

**Abbreviations:** WBCs = white blood cells; RBCs = red blood cells.

* Elevated levels listed if above the reporting laboratory’s reference range.

† Female patient; all others are male.

**TABLE 2:** Results of toxicologic analysis of implicated products and/or clinical specimens from seven patients with acute kidney injury associated with synthetic cannabinoid use — multiple states, 2012

Case no.	State	Implicated product	Synthetic cannabinoids identified from product samples	Clinical specimen type	Days after last use	Synthetic cannabinoids identified from clinical specimens
4	Wyoming	Blueberry-flavored or bubblegum-flavored	XLR-11 and indole precursor	Urine	2	XLR-11 N-pentanoic acid metabolite (400 ng/mL)
				Blood	3	Not detected
6	New York	Phantom Wicked Dreams	Not performed	Blood	2	XLR-11 N-pentanoic acid metabolite (42 ng/mL)
				Blood	3	Not detected
11	Kansas	Mr. Happy	XLR-11 (69 mg/g)	Serum	0	XLR-11 (35 ng/mL); N-pentanoic acid metabolite (102 ng/mL); UR-144 (6 ng/mL)
			UR-144 (61 mg/g)	Urine	0	XLR-11 N-pentanoic acid metabolite (529 ng/mL)
12	Oregon	Clown Loyal	XLR-11 (92.1 mg/g)	Serum	9	Not detected
13	Oregon	Lava	XLR-11 (1.7 mg/g)	Serum	2	XLR-11 (33 ng/mL); N-pentanoic acid metabolite (38 ng/mL)
				Serum	4	Not detected
14	Oregon	Lava	XLR-11 (1.7 mg/g)	Serum	2	Serum insufficient
				Urine	4	Not detected
15	Oklahoma	Flame 2.0	Not detected			Not performed
